# Pre-radiotherapy feeding tube identifies a poor prognostic subset of postoperative p16 positive oropharyngeal carcinoma patients

**DOI:** 10.1186/s13014-014-0314-3

**Published:** 2015-01-09

**Authors:** Vivek Verma, Jingxia Liu, Laura Eschen, Jonathan Danieley, Christopher Spencer, James S Lewis, Jason Diaz, Jay F Piccirillo, Douglas R Adkins, Brian Nussenbaum, Wade L Thorstad, Hiram A Gay

**Affiliations:** Department of Radiation Oncology, University of Nebraska Medical Center, Omaha, Nebraska USA; Division of Biostatistics, Washington University School of Medicine, St. Louis, MO USA; Department of Radiation Oncology, Washington University School of Medicine, 4921 Parkview Place, Campus Box 8224, St. Louis, 63110-6311 Missouri USA; Pathology and Immunology, Washington University School of Medicine, St. Louis, MO USA; Otolaryngology Head and Neck Surgery, Washington University School of Medicine, St. Louis, MO USA; Medical Oncology, Washington University School of Medicine, St. Louis, MO USA

**Keywords:** p16 positive, Oropharynx squamous cell carcinoma, Adjuvant intensity modulated radiation therapy, Definitive surgery, Human papillomavirus, PEG feeding tube

## Abstract

**Background:**

This study explores variables associated with poor prognosis in postoperative p16 positive oropharyngeal squamous cell carcinoma (OPSCC) patients undergoing adjuvant radiotherapy or chemoradiotherapy. Specifically, analysis was done related to timing of feeding tube insertion relative to radiotherapy.

**Methods:**

From 1997–2009, of 376 consecutive patients with OPSCC, 220 received adjuvant IMRT, and 97 were p16 positive and eligible. Of these, 23 had feeding tube placement before IMRT (B-FT), 32 during/after IMRT (DA-FT), and 42 had no feeding tube (NO-FT). Feeding tubes were not placed prophylactically. These three groups were analyzed for differential tumor, patient, treatment, and feeding tube characteristics, as well as differences in overall survival (OS), disease free survival (DFS), and distant metastasis free survival (DMFS).

**Results:**

Pre-RT FT insertion was associated with higher tumor size and depth, T (but not N) and overall stage, comorbidities, presence of chemotherapy, and less use of transoral laser microsurgery/transoral bovie. Additionally, time from surgery to IMRT completion was also statistically longer in the B-FT group. The feeding tube was permanent in 52% of patients in the B-FT group versus 16% in the DA-FT group (p = 0.0075). The 5-year OS for the NO-FT, DA-FT, and B-FT groups was 90%, 86%, and 50%, respectively. The 5-year DFS for the NO-FT, DA-FT, and B-FT groups was 87.6%, 83.6%, and 42.7%, respectively. Multivariate analysis showed that for OS and DFS, feeding tube placement timing and smoking history were statistically significant.

**Conclusion:**

Due to the poor prognosis of early FT insertion, the presence of FTs at time of radiotherapy consultation can be used as an alternate marker to identify a subset of p16 positive OPSCC patients that have a poor prognosis.

## Background

Feeding tube placement for patients with head and neck cancer undergoing radiation therapy (RT) aims to minimize weight loss and maintain nutrition secondary to RT induced mucositis, odynophagia, and/or nausea [1]. Although intensity-modulated radiation therapy (IMRT) attempts to minimize side effects, 50-70% of patients require a feeding tube after chemoradiotherapy, 15-40% with RT alone, and 20-40% with surgery followed by adjuvant RT [2]. In addition to patient comfort, the use of percutaneous endoscopic gastrostomy (PEG) tubes has replaced nasogastric tubes due to better control of weight post-insertion [3].

Prophylactic PEG tube insertion for head and neck cancer patients has been a topic of much controversy [2]. Prophylactic feeding tube proponents note that prophylactic placement not only limits weight loss, especially in advanced unresectable patients [4], but also improves 6-month quality of life [5]. In addition, patients with prophylactically-placed feeding tubes may suffer less morbidity and hospitalizations [6]. On the other hand, prophylactic feeding tube placement has high rates of unnecessary placement (almost 50% per the definitions of Madhoun et al. [7]), and a higher likelihood of prolonged or permanent dependence, especially with advanced T-stage and pre-existing dysphagia [8]. To address these issues, many centers like ours have adopted a “reactive strategy” for feeding tube placement. This plan uses dedicated oral nutritional supplements very early, with strict nutritional follow-up such that if center-dependent nutritional maintenance requirements are not being fulfilled, the patient undergoes feeding tube placement.

In this study we focused on surgically treated p16 positive OPSCC patients who received adjuvant radiotherapy or chemoradiotherapy, a group with overall good prognosis due to human papillomavirus (HPV) related disease. In these patients, feeding tube placement is often assumed to be a consequence of adjuvant radiotherapy or adjuvant chemoradiotherapy, and the prognostic significance of feeding tube placement at various time points is not well understood. The present study identifies a subset of this patient population with a poor prognosis, as related to the timing of percutaneous gastrostomy feeding tube placement.

## Methods

### Patient population

A total of 376 consecutive patients with OPSCC were available for analysis from the years 1997–2009 in our prospective head and neck radiotherapy registry. The diagnosis of SCC was based on report review from the pathology interpreted in routine practice. This included known histologic variants of SCC. Of these patients, 220 underwent postoperative IMRT. Of these 220 patients, p16 status was known in 124 patients, with 104 being p16 positive. Ninety-nine of the p16 positive patients were stage III or IV; two were eliminated due to non-oncologic surgery, which left 97 patients for analysis. Because p16 positive OPSCC generally has a better prognosis than p16 negative disease, we sought to find subgroups of p16 positive OPSCC patients that may do poorly, and thus considered only p16 positive patients. Feeding tubes were not placed prophylactically. Feeding tube placement was left at the discretion of the managing physician (surgeon, medical oncologist, or radiation oncologist), and the decision was based on the patient’s nutritional status and symptoms.

### p16 Immunohistochemistry

Immunohistochemistry was performed on 4 μm sections from formalin-fixed, paraffin-embedded tissue blocks using an antibody to p16 (MTM Laboratories; monoclonal; 1:1 dilution) on a Ventana Benchmark LT automated immunostainer (Ventana Medical Systems, Inc., Tucson AZ) according to standard protocols. Antigen retrieval, standard on the machine, utilized the Ventana CC1, EDTA-Tris, pH 8.0 solution. A known p16 expressing head and neck squamous cell carcinoma or ovarian papillary serous carcinoma case was used as a positive control with each run.

Cases were reviewed independently by the study pathologist without knowledge of the other features of the cases and were classified in quartiles by the extent of cells having both nuclear and cytoplasmic staining as: 0 = no staining; 1+ = 1 to 25% of tumor cells positive; 2+ = 26 to 50%; 3+ = 51 to 75%; 4+ = >76%. Results were divided using a 50% cutoff into negative (0, 1+, or 2+) and positive (3+ or 4+) based on data showing the correlation between extensive p16 expression and the presence of transcriptionally-active HPV [9].

### Surgery

The majority of resectable patients at our institution undergo surgery, preferably transoral laser microsurgery (TLM), so we focused our analysis on p16+ postoperative patients receiving adjuvant radiotherapy, thus providing a unique analysis. Surgical approaches included TLM (57%), as per the principles of Steiner and Ambrosch [10]), transoral bovie approach (21%), or “other” (22%) which included: open mandibulotomy or pharyngotomy, a combination of open and transoral laser or bovie approaches; or transoral ‘cold steel’. The approach was based on surgeon judgment considering the tumor size and location, degree of involvement of nearby structures, and adequacy of transoral access. Neck dissections were generally performed at the time of primary tumor extirpation. Patients underwent appropriate reconstruction when necessary.

### Chemotherapy

Platinum-based chemotherapy (mostly cisplatin 100 mg/m^2^ on days 1, 22, and 43 of RT) was delivered to 43 patients and cetuximab 400 mg/m^2^ loading dose followed by 250 mg/m^2^ weekly × 8 to 3 patients. The use of chemotherapy or cetuximab was based on high-risk pathologic factors such as: ECE, positive margins, pT3/T4, pN2/N3, perineural invasion, and vascular embolism.

### Radiation therapy

Patients were supine and immobilized using a thermoplastic mask. As technology evolved, fusion of PET/CT and/or MRI scans to the planning CT helped define the clinical tumor volumes (CTVs), as well as clinical and pathologic information. In general, CTV1 encompassed the high-risk volume which consisted of the pre-operative primary GTV with a 1.5 cm margin for potential microscopic spread and any involved lymph node levels plus a 0.5 cm to 1 cm margin; CTV2 corresponded to electively treated lymph node levels; and CTV3 consisted of an AP or AP/PA supraclavicular field used at the beginning of our IMRT program. One CTV was defined for 31% of patients, two CTVs for 56% of patients, and three CTVs for 13% of patients. The median dose (range) for CTV1, CTV2, and CTV3 were 66 (60–70) Gy, 56 (54–66.6) Gy and 56 (54 – 56) Gy, respectively. PTVs were defined by adding 0.5 cm to the corresponding CTVs and subtracting 3 mm from the skin.

### Statistical analysis

The primary endpoints were overall survival (OS), disease free survival (DFS), and distant metastases free survival (DMFS). OS was defined as the time from IMRT completion to death from any cause or last follow-up. DFS was defined as the time from IMRT completion to locoregional failure (LRF), distant metastasis or death from any cause, whichever came first. For the patients without any above events, it was defined as the time from IMRT completion to last follow-up. DMFS was defined as the time from IMRT completion to the development of distant metastasis or last follow-up. Duration of feeding tube was defined as the time from feeding tube insertion to removal if the feeding tube was not permanent, and to last follow-up otherwise. Swallow studies were obtained at the discretion of the managing physician(s) after RT for assessment of swallowing dysfunction.

SAS Version 9.3 (Cary, NC) was used to perform all statistical analyses. Continuous and categorical variables were compared by a Kruskal-Wallis test and the Fisher Exact (or chi-square) test, respectively. Kaplan-Meier (KM) curves were generated that provide unadjusted survival estimates for all patients and across strata. Differences between strata were determined by log-rank tests. Univariate and multivariate analyses through Cox proportional-hazards models were considered to evaluate the interested variables for OS, DFS and DMFS. The proportionality assumption was tested by adding a time-dependent covariate for each variable. A backward stepwise model selection approach was performed to identify all significant risk factors. Factors significant at a 10% level were kept in the final model. All statistical tests were two-sided using an α = 0.05 level of significance.

## Results

### Patient, tumor, and management characteristics

Tables 1, 2, and 3 summarize the patient and tumor characteristics for the NO-FT (43% of patients), B-FT (24%), and DA-FT (33%) groups. Of note, the feeding tube was permanent in 52% of patients in the B-FT group versus 16% in the DA-FT group (p = 0.0075). The locoregional relapse rate for the entire group was 2%. The two patients who failed only received adjuvant radiotherapy versus adjuvant chemoradiotherapy.

**Table 1:** **Patient characteristics for the three patient groups**

**Characteristic**	**No feeding tube (n = 42)**	**Tube before RT (n = 23)**	**Tube during/after RT (n = 32)**	**p-value**
Age at diagnosis				
Median (range) (y)	54 (32 – 73)	57 (48 – 67)	54 (37 – 72)	0.35
Gender				
Male	40 (95.2%)	21 (91.3%)	31 (96.9%)	0.72
Female	2 (4.8%)	2 (8.7%)	1 (3.1%)	
Race				
Caucasian	40 (95.2%)	22 (95.7%)	32 (100%)	0.46
African-American	2 (4.8%)	1 (4.3%)	0 (0%)	
Alcohol Use				
Rarely or Never	22 (56.4%)	13 (56.5%)	17 (53.1%)	0.51
Occasional	8 (20.5%)	3 (13.0%)	10 (31.3%)	
Heavy	9 (23.1%)	7 (30.4%)	5 (15.6%)	
Smoking Status				
Never Smoker	21 (51.2%)	8 (34.8%)	9 (28.1%)	0.29
<20 pack-years	5 (12.2%)	4 (17.4%)	4 (12.5%)	
≥20 pack-years	15 (36.6%)	11 (47.8%)	19 (59.4%)	
ACE-27 Score				
None/Mild	38 (95.0%)	15 (68.2%)	23 (74.2%)	**0.0076**
Moderate/Severe	2 (5.0%)	7 (31.8%)	8 (25.8%)	
BMI at time of tube placement				
Median (range)	N/A	26.5 (20.0 – 31.0)	24.5 (15.7 – 41.2)	0.12
Rate of weight loss prior to IMRT (− lbs/mo loss; + is gain)				
Median (range)	−6.9 (−19.0 – +5.0)	−10.6 (−20 – +0.6)	−1.2 (−12 – +2.8)	0.36
Swallow Studies				
Aspiration	10 (76.9%)	18 (85.7%)	14 (77.8%)	0.74
No aspiration	3 (23.1%)	3 (14.3%)	4 (22.2%)	

*Abbreviations*: *ACE-27* Adult Comorbidity Evaluation-27.

Bold p-values indicate statistical significane at p < 0.05.

**Table 2:** **Tumor characteristics for the three patient groups**

**Characteristic**	**No feeding tube (n = 42)**	**Tube before RT (n = 23)**	**Tube during/after RT (n = 32)**	**p-value**
Tumor Location				
Tonsil	23 (54.8%)	8 (34.8%)	18 (56.3%)	0.23
Base of Tongue	18 (42.9%)	12 (52.2%)	11 (34.4%)	
Other	1 (2.4%)	3 (13.0%)	3 (9.4%)	
Tumor Laterality				
Right	21 (50.0%)	9 (39.1%)	17 (53.1%)	0.67
Left	18 (42.9%)	11 (47.8%)	14 (43.8%)	
Bilateral	3 (7.1%)	3 (13.0%)	1 (3.1%)	
Perineural Invasion				
Yes	3 (8.8%)	7 (31.8%)	4 (14.3%)	0.089
No	31 (91.2%)	15 (68.2%)	24 (85.7%)	
Vascular Invasion				
Yes	10 (27.0%)	8 (25%)	3 (11.1%)	0.095
No	27 (73.0%)	14 (75%)	24 (88.9%)	
Lymphatic Invasion				
Yes	16 (43.2%)	15 (68.2%)	10 (34.5%)	0.054
No	21 (56.8%)	7 (31.8%)	19 (65.5%)	
Extracapsular Extension				
Positive	35 (87.5%)	21 (91.3%)	26 (81.3%)	0.56
Negative	5 (12.5%)	2 (8.7%)	6 (18.7%)	
Soft Tissue Metastases				
Yes	20 (64.5%)	14 (66.7%)	17 (65.4%)	1.00
No	11 (35.5%)	7 (33.3%)	9 (34.6%)	
Tumor Size (cm)				
Median (range)	2.0 (0.2 – 6.0)	4.0 (1.2 – 12.0)	2.25 (0.5 – 6.6)	**0.0014**
Tumor Depth (cm)				
Median (range)	0.75 (0.05 – 5.0)	1.2 (0.45 – 4.5)	0.65 (0.12 – 1.9)	**0.014**
Pathologic T stage				
T1	23 (54.8%)	2 (8.7%)	13 (40.6%)	**<0.0001**
T2	14 (33.3%)	5 (21.7%)	17 (53.1%)	
T3	3 (7.1%)	6 (26.1%)	2 (6.3%)	
T4a	2 (4.8%)	10 (43.5%)	0 (0%)	
Largest Lymph Node (cm)				
Median (range)	3.0 (1.3 – 6.0)	3.7 (0.7 – 6.0)	3.3 (0.6 – 7.5)	0.67
Total Number of Metastatic Lymph Nodes				
Median (range)	2 (1 – 15)	4 (1 – 25)	2 (1 – 40)	0.19
Pathologic N stage				
N1	9 (21.4%)	2 (8.7%)	7 (21.9%)	0.072
N2a	9 (21.4%)	4 (17.4%)	2 (6.3%)	
N2b	20 (47.6%)	11 (47.8%)	16 (50.0%)	
N2c	4 (9.5%)	6 (26.1%)	3 (9.4%)	
N3	0 (0%)	0 (0%)	4 (12.5%)	
Pathologic stage				
III	9 (21.4%)	1 (4.3%)	7 (21.9%)	**0.016**
IVA	33 (79.6%)	22 (95.7%)	21 (65.6%)	
IVB	0 (0%)	0 (0%)	4 (12.5%)	

Bold p-values indicate statistical significane at p < 0.05.

**Table 3:** **Management characteristics for the three patient groups**

Type of Surgery				
Transoral Laser	28 (66.7%)	6 (26.1%)	21 (65.6%)	**<0.0001**
Transoral Bovie	9 (21.4%)	1 (4.3%)	10 (31.3%)	
Other*	5 (11.9%)	16 (69.6%)	1 (3.1%)	
Margins				
Positive	4 (10.0%)	3 (13.6%)	4 (13.8%)	0.85
Negative or < 5 mm	36 (90.0%)	19 (86.4%)	25 (86.2%)	
Chemotherapy				
Yes	12 (28.6%)	14 (56.0%)	20 (62.5%)	**0.0050**
No	30 (71.4%)	9 (44.0%)	12 (37.5%)	
Time from Surgery to Completion of RT (days)				
Median (range)	91 (74 – 135)	107 (85 – 140)	92 (81 – 144)	**0.0008**
Duration of feeding tube (mo)				
Median (range)	N/A	7.27 (3.91 – 40.53)	5.1 (1.68 – 22.96)	0.069
Was feeding tube permanent?				
Yes	N/A	12 (52.2%)	5 (16.1%)	**0.0075**
No	N/A	11 (47.8%)	26 (83.9%)	
Cause of Death				
Locoregional Disease	0 (0%)	2 (15.4%)	0 (0%)	0.36
Distant Metastatic Disease	3 (60%)	1 (7.7%)	2 (33.3%)	
Second Primary	1 (20%)	5 (38.5%)	3 (50%)	
All Other Causes	1 (20%)	5 (38.5%)	1 (16.7%)	

*Abbreviations*: *TLM* transoral laser microsurgery.

*One of the following: open mandibulotomy; open pharyngotomy; open pharyngotomy and transoral CO2 laser; open pharyngotomy and transoral bovie; open mandibulotomy and transoral CO2 laser; open pharyngotomy; and transoral ‘cold steel’.

Bold p-values indicate statistical significane at p < 0.05.

Feeding tube placement was associated with the type of surgery, pathologic T stage, pathologic stage, ACE-27 score, and chemotherapy. Patients in the B-FT group had the highest percentage of patients with “other” type of surgeries (69.6%), T4a tumors (43.5%), stage IVa tumors (95.7%), and moderate/severe ACE-27 scores (31.8%). There was a statistically significant difference in the median tumor size, depth, and time from surgery to completion of IMRT among the three groups. Patients in the B-FT group had the largest median tumor size (4 cm) and depth (1.2 cm), and longest median time to complete IMRT (107 days).

Feeding tube placement was not associated with gender, race, alcohol use, smoking status, margin status, tumor laterality and location, perineural invasion, vascular invasion, lymphatic invasion, extracapsular extension, soft tissue metastases, N stage, or aspiration observed in a swallow study. There was no statistically significant difference in age, BMI at time of tube placement, rate of weight loss prior to RT, or duration of feeding tube among groups.

### Kaplan-Meier plot

**Figure 1:**
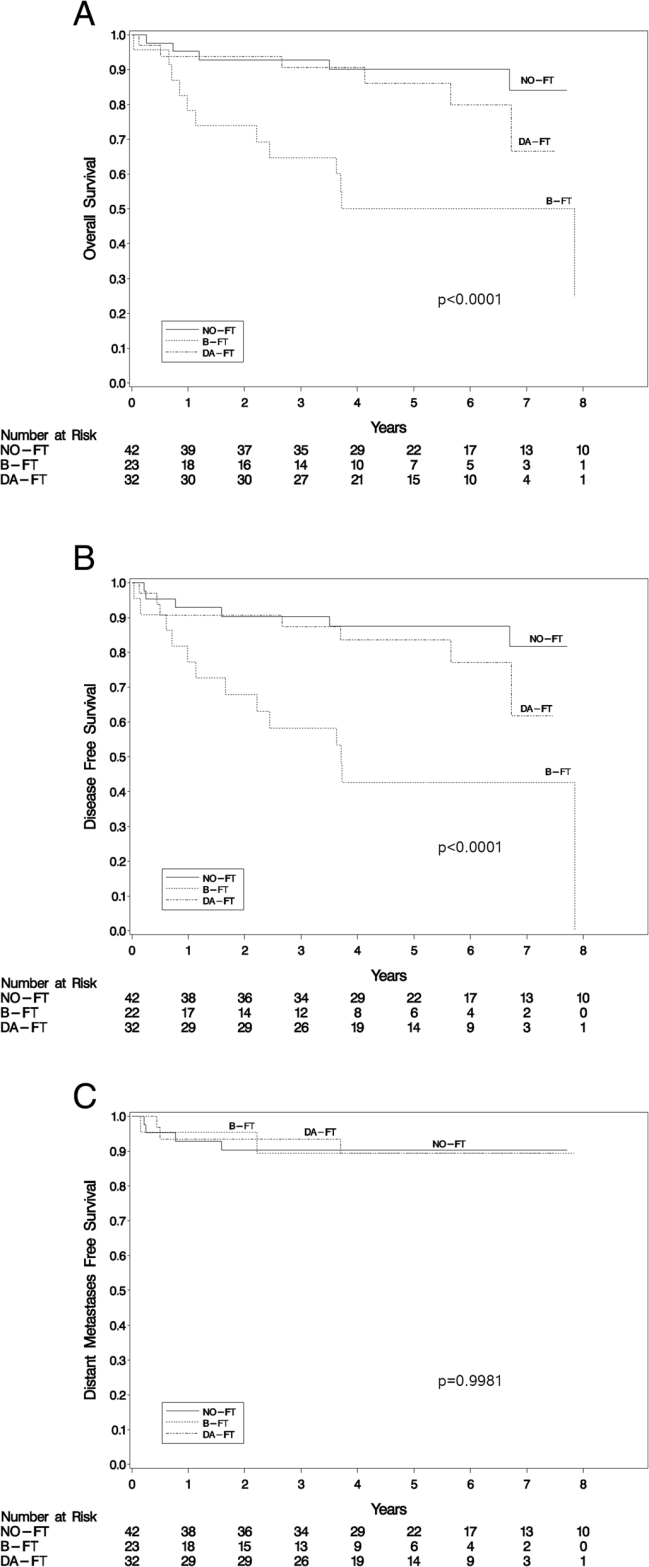
**Overall survival (A), disease free survival (B), and distant metastases free survival (C) for the no feeding tube (NO-FT), before IMRT feeding tube (B-FT), and during or after IMRT feeding tube (DA-FT).**

There was a statistically significant difference in OS (Figure 1A) and DFS (Figure 1B) among the three groups (p <0.0001 for both), but not in DMFS (Figure 1C) (p = 0.9981). The 5-year OS for the NO-FT, DA-FT, and B-FT groups was 90%, 86%, and 50%, respectively. The 5-year DFS for the NO-FT, DA-FT, and B-FT groups was 87.6%, 83.6%, and 42.7%, respectively. The 5-year DMFS for the NO-FT, DA-FT, and B-FT groups was 90.3%, 89.5%, and 89.5%, respectively. Additionally, there was a statistically significant difference (p < 0.0001) if the NO-FT and DA-FT groups were combined and compared to the B-FT group in terms of both OS and DFS.

### Univariate analysis

On univariate analysis through Cox-proportional hazards models (Table 4), significant predictors of DFS and OS on univariate analysis included: feeding tube group, age, smoking status, and type of surgery performed. T-stage and ACE-27 score were significant factors for OS only.

**Table 4:** **Univariate analysis for overall survival, disease free survival, and distant metastases**

**Overall survival**	**p-value**
FT Group	
B-FT vs. NO-FT	**0.0003**
DA-FT vs. NO-FT	
Age (years)	
≥50 vs. < 50	**0.0435**
Smoking	
<20 pack-years vs. never smoker	**0.0272**
≥20 pack-years vs. never smoker	
Type of Surgery	
TLM vs. other	0.0024*
transoral bovie vs. other	
ACE Score	
Moderate/ Severe vs. Mild/None	0.0253*
Combined T Stage	
T1 vs. T3/T4	0.0440*
T2 vs. T3/T4	
**Disease Free Survival**	
FT Group	
B-FT vs. NO-FT	**0.0004**
DA-FT vs. NO-FT	
Age (years)	
≥50 vs. < 50	**0.0374**
Smoking	
<20 pack-years vs. never smoker	**0.0346**
≥20 pack-years vs. never smoker	
Type of Surgery	
TLM vs. other	0.0024*
transoral bovie vs. other	
ACE Score	
Moderate/Severe vs. Mild/None	0.0675*
Combined T Stage	
T1 vs. T3/T4	0.0509*
T2 vs. T3/T4	
**Distant Metastases Free Survival**	
Group	
B-FT vs. NO-FT	0.9981
DA-FT vs. NO-FT	
Age (years)	
≥50 vs. < 50	0.1060

*Abbreviations*: *B-FT* Feeding tube placement before IMRT, *DA-FT* Feeding tube placement during or after IMRT, *NO-FT* No feeding tube, *TLM* Transoral laser microsurgery, *CI* Confidence interval.

*Significant in univariate but not in multivariate analysis.

### Multivariate analysis

As shown in Table 5, the only predictors of both OS and DFS on multivariate analysis were feeding tube group and smoking status. Specifically, the B-FT group had a 6.0 HR (2.0 - 18.6, 95% CI) for OS and 4.7 HR (1.7 - 13.1, 95% CI) for DFS when compared to the NO-FT group. The ≥ 20 pack-years smoking group had a 5.0 HR (1.6 - 15.3, 95% CI) for OS and 3.8 HR (1.4 - 10.6, 95% CI) for DFS when compared to the never smoker group.

**Table 5:** **Multivariate analysis for overall survival, disease free survival, and distant metastases**

**Overall survival**	**HR (95% CI)**	**p-value**
FT Group		
B-FT vs. NO-FT	**6.0 (2.0 – 18.6**)	**0.0013**
DA-FT vs. NO-FT	1.5 (0.4 – 5.4)	
Age (years)		
≥50 vs. < 50	2.1 (0.8 – 5.3)	0.1165
Smoking		
<20 pack-years vs. never smoker	3.6 (0.8 – 16.9)	**0.0182**
≥20 pack-years vs. never smoker	**5.0 (1.6 – 15.3)**	
**Disease Free Survival**		
FT Group		
B-FT vs. NO-FT	**4.7 (1.7 – 13.1)**	**0.0017**
DA-FT vs. NO-FT	1.3 (0.4 – 4.0)	
Age (years)		
≥50 vs. < 50	2.3 (1.0 – 5.4)	0.0619
Smoking		
<20 pack-years vs. never smoker	2.2 (0.5 – 9.5)	**0.0316**
≥20 pack-years vs. never smoker	**3.8 (1.4 – 10.6)**	
**Distant Metastases Free Survival**		
Group		
B-FT vs. NO-FT	0.91 (0.17 – 5.02)	0.9942
DA-FT vs. NO-FT	0.95 (0.21 – 4.27)	
Age (years)		
≥50 vs. < 50	3.7 (0.8 – 17.8)	0.1053

*Abbreviations*: *B-FT* Feeding tube placement before IMRT, *DA-FT* Feeding tube placement during or after IMRT, *NO-FT* No feeding tube, *TLM* Transoral laser microsurgery, *CI* Confidence interval.

### Causes of death

At last follow-up, 24 (25%) patients had died. The causes of death in the B-FT group were as follows: locoregional disease, 2 (9%); distant metastases, 1 (4%); second primary, 5 (22%); and 5 (22%) of all other causes. The causes of death in the DA-FT group were as follows: distant metastases, 2 (6%); second primary, 1 (3%); and 3 (9%) of all other causes. The causes of death in the NO-FT group were as follows: distant metastases, 3 (7%); second primary, 1 (2%); and 1 (2%) of all other causes. Out of the seven patients with second primary tumors, five were lung cancer, one esophageal cancer, and one with auditory canal squamous cell carcinoma.

### Reasons for feeding tube placement

The physician decision to place a feeding tube has both objective and subjective criteria based on clinical experience. Our policy is to delay feeding tubes as clinically feasible without compromising the patients’ health. At our center, the patients’ weight is monitored weekly. Patients are also evaluated by a nutritionist weekly. If the patients’ weight drop is between 5 to 10%, or if patients experience symptoms such as not being able to eat and/or drink for more than 24 hours, the reasoning for FT placement is mostly due to poor PO intake. Other more subjective patient factors such as the severity of dysphagia and/or odynophagia also influenced the decision. Based on these factors the physician has a conversation regarding feeding tube placement. Some patients agree at this point to proceed with a feeding tube, while others opt to delay the feeding tube hoping they can increase their caloric intake. Patients who delay the feeding tubes often have further drops in weight.

Poor nutritional status was the most common reason for feeding tube placement in the B-FT group (77.3%), followed by dysphagia alone (9.1%), poor PO intake (9.1%), and dysphagia + poor nutritional status (4.5%). Poor PO intake was the most common reason for feeding tube placement in the DA-FT group (28.1%), followed by dysphagia (18.8%), dysphagia + odynophagia + poor PO intake (15.6%), dysphagia + poor PO intake (9.4%), dysphagia + weight loss (9.4%), dysphagia + odynophagia + poor PO intake + weight loss (9.4%), poor PO intake + weight loss (3.1%), odynophagia + poor PO intake + weight loss (3.1%), and weight loss alone (3.1%).

## Discussion

Patients with p16 positive OPSCC have a relatively good prognosis [11]. However, in our 97 patient group, we were able to identify a subset of 23 patients who had the feeding tube placed prior to IMRT (of note, we followed a reactive rather than prophylactic feeding tube placement policy which is important when interpreting the results). These patients had a significantly worse prognosis with a 50% 5-year overall survival, compared to 88.3% for the rest of the group. In this patient population, T-stage has been a significant factor for OS and DFS [12,13]. However, in our multivariate analysis, feeding tube grouping was significant, while T stage was not. The reason for this is not clear, but it may be that the poor prognosis B-FT group has more poor-prognostic variables. Specifically, patients in the B-FT group had the highest percentage of patients with “other” type of surgeries (requiring open approaches versus exclusively minimally invasive approaches), T4a tumors, stage IVA tumors, and moderate/severe ACE-27 scores. Our results are hence consistent with previous data that feeding tubes are more likely to be inserted in patients with more advanced disease and comorbidities [14].

Patients in the B-FT group also had the largest median tumor size and depth, and longest median time to complete IMRT, 107 days (more than two weeks compared to the median of the other 2 groups). An overall treatment length ≥ 100 days has been associated with a worse local control and survival [15]. Similarly, the median (range) days from surgery to start of IMRT was longest for the B-FT group at 57 (34 – 94) days, compared to the NO-FT group at 46 (30 – 84) days and the DA-FT group at 44.5 (32 – 93) days. However, in this study only 2 patients died from locoregional disease, which does not support the hypothesis that longer treatment times or higher T stage, variables associated with a higher risk of local recurrence, explain the worse prognosis of the B-FT group.

The magnitude of these patient and tumor characteristics or delay in IMRT completion in treatment outcomes could have different implications. If the delay in IMRT completion has the biggest impact, then surgery may be suboptimal for some of these patients. Although a direct comparison is not possible between the B-FT group and non-surgical series, the 3-year OS [16,17] in predominantly definitively treated patients with IMRT has ranged between 78 and 83%, compared to the 64.7% 3-year OS in the B-FT group.

Contrary to popular belief, 24% of patients had a feeding tube inserted prior to IMRT, while 33% of patients had it during or after IMRT. In addition, the feeding tube was permanent in 52% of patients who had the feeding tube inserted prior to IMRT, versus 16% in the DA-FT group. Patients with a feeding tube at the time of radiation oncology consult should be counseled accordingly.

Another implication of this study is that because the presence of a pre-RT FT was associated with poor prognostic factors and variables associated with a higher risk of FT placement (such as comorbidities, T and overall stage, chemotherapy), the presence of a FT at time of radiation oncology consultation can be effectively used as a quick but reliable indicator of worse disease status in patients with p16 positive OPSCC. This use of a surrogate marker should alert clinicians that the patient is part of a higher-risk subgroup of p16 positive OPSCC with relatively worse prognosis.

The B-FT group did not have a higher risk of aspiration (p = 0.74) during swallowing studies compared to the other groups which could have explained the increase in mortality. There was no statistically significant difference (p = 0.36) in the cause of death among the 3 groups. This suggests that the feeding tube insertion prior to IMRT is of prognostic significance and not responsible for the increased mortality in this group. One area of further investigation will be to predict which patients have the highest risk of requiring a feeding tube at the time of surgery.

## Conclusion

Due to the poor prognosis of early FT insertion, the presence of FTs at time of radiotherapy consultation can be used as an alternate marker to identify a subset of p16 positive OPSCC patients that have a relatively poor prognosis.
